# Asian Population Is More Prone to Develop High-Risk Myelodysplastic Syndrome, Concordantly with Their Propensity to Exhibit High-Risk Cytogenetic Aberrations

**DOI:** 10.3390/cancers13030481

**Published:** 2021-01-27

**Authors:** Yan Jiang, Jean-Richard Eveillard, Marie-Anne Couturier, Benoit Soubise, Jian-Min Chen, Sujun Gao, Audrey Basinko, Frédéric Morel, Nathalie Douet-Guilbert, Marie-Bérengère Troadec

**Affiliations:** 1 Univ Brest, Inserm, EFS, UMR 1078, GGB, F-29200 Brest, France; jiangyanjdyy@jlu.edu.cn (Y.J.); benoit.soubise@hotmail.fr (B.S.); jian-min.chen@univ-brest.fr (J.-M.C.); frederic.morel@univ-brest.fr (F.M.); Nathalie.douet-guilbert@chu-brest.fr (N.D.-G.); 2 Department of Hematology, The First Hospital of Jilin University, Changchun 130021, China; sjgao@jlu.edu.cn; 3 CHRU Brest, Service d’Hématologie, F-29200 Brest, France; jean-richard.eveillard@chu-brest.fr (J.-R.E.); marie-anne.couturier@chu-brest.fr (M.-A.C.); 4 CHRU Brest, Service de Génétique, Laboratoire de Génétique Chromosomique, F-29200 Brest, France; audrey.basinko@chu-brest.fr

**Keywords:** myelodysplastic syndrome, cytogenetics, prognosis, survival, Europe, Asia, treatments, del(5q), trisomy 8, del(20q), *TET2*, *SF3B1*, *SRSF2*, *IDH1/2*, *RUNX1*, *U2AF1*, *ETV6*

## Abstract

**Simple Summary:**

The world population is genetically and environmentally diverse. In particular, genetic differences related to an ethnic factor may underlie differences in cancer phenotypic expression. Therefore, we compared the epidemiology, and the clinical, biological and genetic characteristics of myelodysplastic syndrome (MDS) between Asian and Western countries. Our results show substantial differences in the incidence and age of onset between Asian and Western MDS patients. A higher proportion of Asian MDS patients fall into the high- and very-high risk prognostic MDS groups. This finding is supported by the identification of a higher proportion of high-risk cytogenetic aberrations in Asian MDS patients. However, the survival rate is similar for Western and Asian MDS patients. Our findings may impact the clinical management as well as the strategy of clinical trials targeting those genetic aberrations and mutations depending on the world area where they are run.

**Abstract:**

This study explores the hypothesis that genetic differences related to an ethnic factor may underlie differences in phenotypic expression of myelodysplastic syndrome (MDS). First, to identify clear ethnic differences, we systematically compared the epidemiology, and the clinical, biological and genetic characteristics of MDS between Asian and Western countries over the last 20 years. Asian MDS cases show a 2- to 4-fold lower incidence and a 10-year younger age of onset compared to the Western cases. A higher proportion of Western MDS patients fall into the very low- and low-risk categories while the intermediate, high and very high-risk groups are more represented in Asian MDS patients according to the Revised International Prognostic Scoring System. Next, we investigated whether differences in prognostic risk scores could find their origin in differential cytogenetic profiles. We found that 5q deletion (del(5q)) aberrations and mutations in *TET2, SF3B1*, *SRSF2* and *IDH1/2* are more frequently reported in Western MDS patients while trisomy 8, del(20q), *U2AF1* and *ETV6* mutations are more frequent in Asian MDS patients. Treatment approaches differ between Western and Asian countries owing to the above discrepancies, but the overall survival rate within each prognostic group is similar for Western and Asian MDS patients. Altogether, our study highlights greater risk MDS in Asians supported by their cytogenetic profile.

## 1. Introduction

Myelodysplastic syndromes (MDS) are acquired clonal stem cell disorders characterized by inefficient hematopoiesis, refractory cytopenia and appreciable risk of progression to acute myeloid leukemia [1]. MDS are very heterogeneous for their morphology, clinical features, and the survival of patients.

Recent advances on MDS have been achieved by targeted therapies. Isolated del(5q) MDS patients are more responsive to lenalidomide [2,3,4]. Moreover, clinical trials are running with drugs targeting *TP53* mutation or *IDH1/IDH2* mutations [5,6]. To anticipate the world-wide use of those new targeted therapies and appreciate the range of their use, we decided to explore whether ethnic differences are observed in MDS presentation and, if appropriate, whether those differences are supported by genetic differences. In order to simplify the question, we decided to focus on two parts of the world: The Western population including America and Europe, and the Asian population. Indeed, several studies, mainly Asian [7,8,9] ones, report some differences in clinical features between Western and Asian MDS patients. However, till now, no systematic reports about these variations have been published, and their implications not yet fully considered. The current paradigms for diagnosis and treatments are mainly based on Western data, which may not be entirely appropriate for Asian populations in clinical practice. 

## 2. Results and Discussion

### 2.1. The Age of Onset Is Earlier and the Incidence Lower in Asia Compared to Western Population

**Figure 1:**
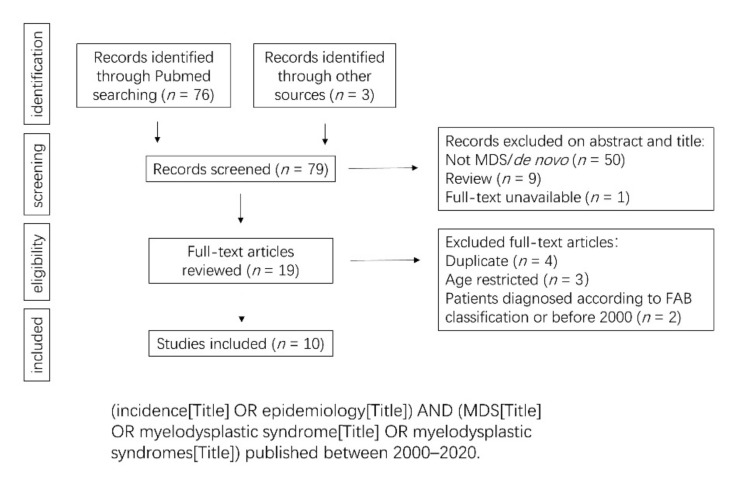
Flow chart for the process of identifying studies included in and excluded from the systematic review for the comparisons of MDS incidence and epidemiology data.

To compare the epidemiological data from Western and Asian countries for the past 20 years, we follow the flow chart presented in Figure 1. A Pubmed search was done on MESH terms and specific criteria (incidence, epidemiology, myelodysplastic syndrome, MDS, publication between 2000–2020). We also included data from the national reference registries of MDS. After exclusion of some articles, we based our study on 10 articles [10,11,12,13,14,15,16,17,18,19] (Figure 1).

#### 2.1.1. Incidence and Age-Adjusted Incidence

**Figure 2:**
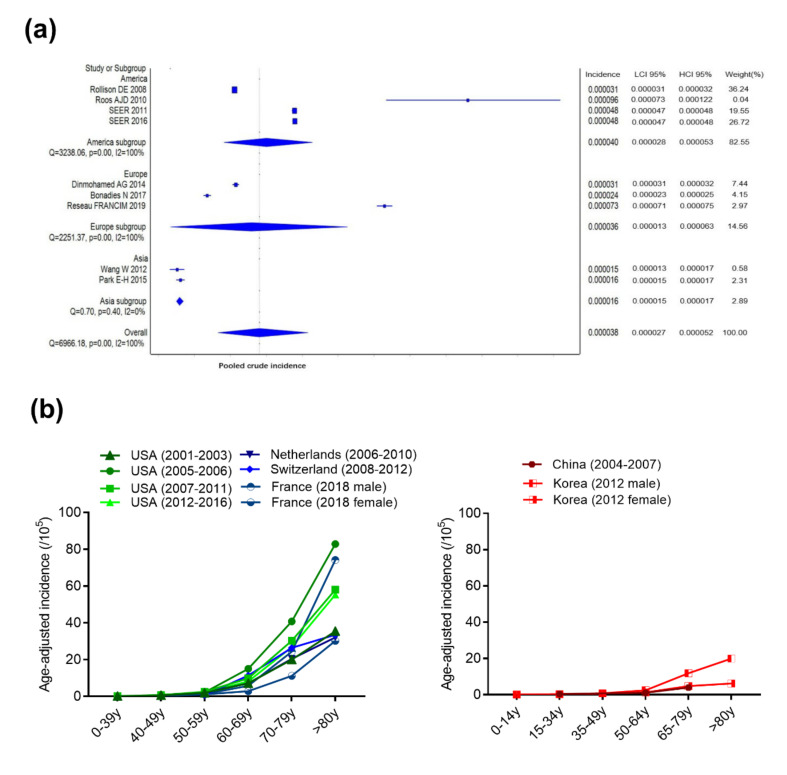
Epidemiological characteristics of MDS in Western and Asian countries. (**a**) Pooled crude incidence in America, Europe, and Asia. (**b**) Age-adjusted incidence in Western and Asian countries. Figure 2 has been drawn based on the data and publications [10,11,12,13,14,15,16,17,18,19] described in Supplemental Table S1.

The overall incidence for the Asian subgroup is 1.6 cases per 100,000 inhabitants/year (95% confidence interval (CI): 1.5–1.7) (Figure 2a). On the contrary, the incidence rises to 3.6 cases per 100,000 inhabitants/year in the European subgroup (95% CI: 1.3–6.3) and 4.0 cases per 100,000 inhabitants/year in the American subgroup (95% CI: 2.8–5.3) (Figure 2a). The differences of incidence between Asian, European and American countries are statistically significant (*p* < 0.0001). More precisely, the raw data on reported age-adjusted incidence ranges from 1.13–1.51 cases per 100,000 inhabitants/year for independent studies in Asian countries to 2.26–7 cases per 100,000 inhabitants/year for independent studies in Western countries (Figure 2b, Table S1). 

The diagnostic criteria have evolved over the past two decades; therefore, the incidence reported along different time periods may vary even within the same region of the world. From the same resource, the incidence reported for the United States from 2001 to 2016, the Netherlands from 2001 to 2010, and Switzerland from 2001 to 2012 shows a slight increase, which is mainly attributed to the elderly group [10,13,14,15] (Table S1, Figure 2b). 

The incidence of MDS is higher in Western countries compared to Asian ones. For instance, when considering the year 2012, the incidence of MDS in Western countries is still 2- to 4-fold higher than that of Korean patients [13,15,19] (Table S1, Figure 2b).

Innate and acquired causes can explain such a discrepancy of the MDS incidence between Western and Asian populations: different genetic susceptibilities among ethnic groups, geographical and dietary reasons, occupational and environmental stresses among which toxin exposures, such as benzene, insecticide and cigarette smoke [20], virus infection [8], previous therapeutic treatments, such as chemotherapy and radiotherapy, and finally, suboptimal case ascertainment and underreporting [1]. For instance, in the United States, the incidence of MDS within the “White” people population is higher than that of Asian, Pacific Islander, American Indian, and Alaska Native people [10,13].

#### 2.1.2. Age of Onset

MDS occurs mainly in the elderly; the median age at diagnosis is ~76 years old in Europe (Figure 2b, Table S1). The data from French register, 2018 FRANCIM, indicates the median age in France to be 78 years old for men and 80 years old for women [16] (Figure 2b, Table S1). By contrast, the median age in China and Japan is earlier than that of Western countries, ranging from 62–76 years old [17,18]. Punctually, a median age of 57 years old has been once reported in a Chinese study [21], and another Chinese report from 2013 showed that over half of the patients with MDS (61.9%) were younger than 60 year-old at the first diagnosis [22]. By contrast, about 10% of the patients were younger than 50 years of age in a German study from 2011 [23]. The causes explaining such a difference in the age of onset between Asian and Western countries are still unclear. The use of different diagnostic criteria, and genetic and environmental factors may be part of the explanation, in addition to differences in percentage of older population and life expectancy between different countries [18,24]. In particular, genetic factors may have their importance in those differences. This specific point will be investigated in Section 2.4.

#### 2.1.3. Gender Ratio

A clear male predominance in MDS patients exists both in Western and Asian countries; the reported gender ratio is 1.24–2.125. The difference tends to be more significant in patients older than 60 years of age, based on the French and the Korean national cancer registries (Figure 2b, Table S1). In patients older than 80 years old, the incidence of males with MDS is three times higher than that of females [19]. More female patients have been once reported in a Chinese study, but a high percentage of young patients could have been responsible for this result [18]. Interestingly, the female prevalence is more significant in 5q- syndrome presenting the del(5q) as the sole abnormality, but the reason for this female representation is still unknown [25].

Altogether, MDS is less frequent in Asia compared to Western population, but the disease may be more severe as it appears earlier. To go further, we analyzed the distribution of MDS subtypes. 

### 2.2. Asian Population is More Prone to Develop Severe MDS Subtypes

#### 2.2.1. Subtype Distribution

**Figure 3:**
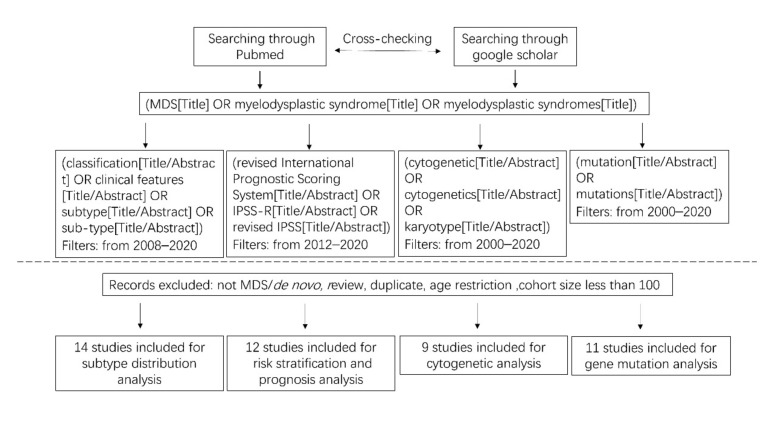
Flow chart for the process of identifying studies included in and excluded from the systematic review for the comparisons of MDS subtypes, risk stratification and prognosis, cytogenetic abnormalities, and gene mutation.

**Figure 4:**
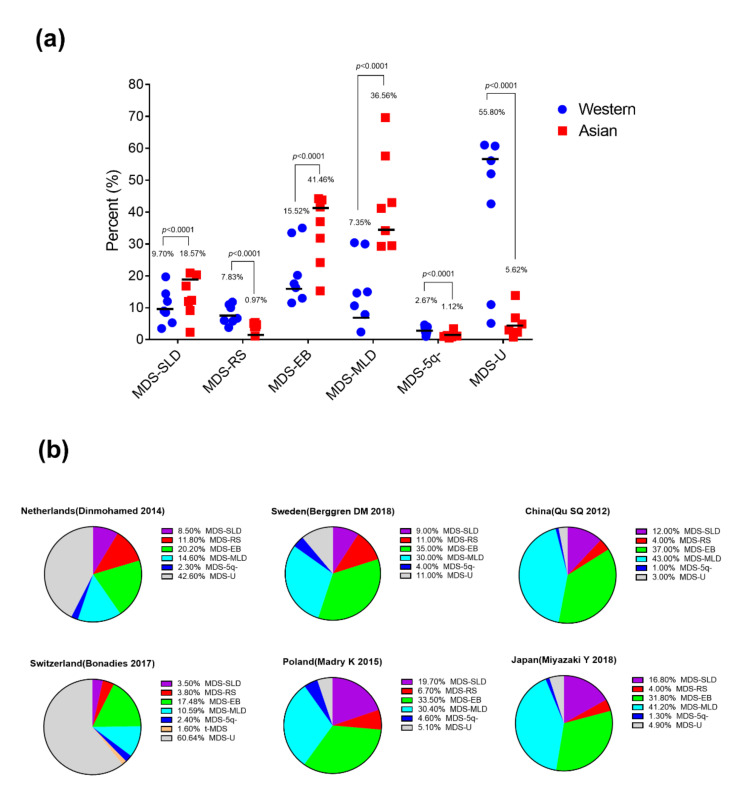
MDS subtypes in Western and Asian countries. (**a**,**b**), Percentage of MDS subtypes in Western and Asian countries. Figure 4 has been drawn based on the data and publications [10,13,14,15,18,19,21,24,26,27,28,29,30,31] described in Supplemental Tables S2 and S3. Importantly, the numbers indicated above each condition correspond to the weighted average (taking into account the size of the cohort of each study).

We collected the data according to the 2008 World Health organization (WHO) classification and the International Classification of Diseases for Oncology (ICD-O) [10,13,14,15,18,19,21,24,26,27,28,29,30,31] (Figure 3 and Figure 4a, Tables S2 and S3). Collectively, the sizes of Western and Asian cohorts are respectively 63,394 and 9675 patients (Table S3).

The frequency of MDS with single lineage dysplasia (MDS-SLD) among all MDS subtypes is statistically higher in Asian countries compared to Western countries (18.57% vs. 9.70%, *p* < 0.0001). 

The data show statistically lower percentages of MDS with ring sideroblasts (MDS-RS) (0.97% vs. 7.83%, *p* < 0.0001) and MDS with isolated del(5q) (MDS-5q-) (1.12% vs. 2.67%, *p* < 0.0001) in Asian countries compared to Western countries. MDS-RS and MDS with isolated del(5q) mostly occur in old patients and comprise good- and intermediate- cytogenetic risk [32]. The high proportion of these two subtypes may be a reason explaining that older patients are more frequently found in Western countries [15]. However, the 2016 WHO classification revised its criteria from the previous 2008 WHO classification for MDS with isolated del(5q). The revised classification defines MDS with isolated del(5q) even when 1 additional cytogenetic abnormality is also present besides the del(5q), excepting monosomy 7 or del(7q) [33]. Upon the revised criteria, the subtype distribution has changed. As most of the del(5q) aberrations in Asian population are accompanied by other chromosomal abnormalities [29], the percentage of MDS with isolated del(5q) in Asian countries would increase more significantly.

More MDS cases with excess of blasts (MDS-EB) are statistically present in the Asian studies compared to Western ones (41.46% vs. 15.52%, *p* < 0.0001).

More strikingly, higher percentages of MDS with multilineage dysplasia (MDS-MLD) are reported in Asian countries compared to Western countries (36.56% vs. 7.35%, *p* < 0.0001). 

By contrast, MDS unclassifiable (MDS-U) are rarely reported in the Asian countries compared to European and North American studies (5.62% vs. 55.80%, *p* < 0.0001). However, it is noteworthy that the data from two European countries, Poland and Sweden, are similar to the Asian results (Figure 4b). The geographical location and past oriental migrations are considered plausible explanations for these similarities [34]. Besides, a study from Mayo clinic evaluated the patients who met WHO criteria for MDS at their institution. In this study, ninety patients (11%) were initially classified as MDS-U, and after pathological review, only half of the cases were confirmed to be MDS-U, while the other half were reclassified to another subtype by follow-up [35]. Making the MDS diagnosis at an early stage of the disease may also be a reason explaining that more MDS-U subtype is reported in European and North American countries compared to Asian ones.

Finally, rare literature data exists in terms of t-MDS subtype. As more patients survive primary malignancies after receiving intensive chemotherapy and/or radiation, the proportion of t-MDS is likely to increase. Still, the difference of t-MDS between Western and Asian countries needs more evidence to be asserted.

#### 2.2.2. Cell Morphology

In the past 30 years, a noticeable discrepancy has existed in various reports due to heterogeneous diagnostic criteria. However, a study from 2005 reported that a significant concordance is achieved when using the same classification [7]. In this study, the morphological diagnosis between Japanese and German hematologists was 98.4% concordant according to FAB classification, and 83.8% concordant with the WHO classification. Interestingly, still from this study and under the same classification, Asian patients compared to the patients in Western countries present a more pronounced cytopenia, especially more severe thrombocytopenia, and a higher frequency of pancytopenia or bicytopenia [7]. 

Altogether, those results show that Asian MDS patients present more frequently MDS considered as severe phenotype (MDS-EB, MDS-MLD, more pronounced cytopenia, higher frequency of pancytopenia or bicytopenia). To further explore the severity of MDS, we analyzed the prognostic score.

### 2.3. Asian MDS Patients Fall More Frequently into the Intermediate-, High- and Very High-Risk Pronostic Groups

**Figure 5:**
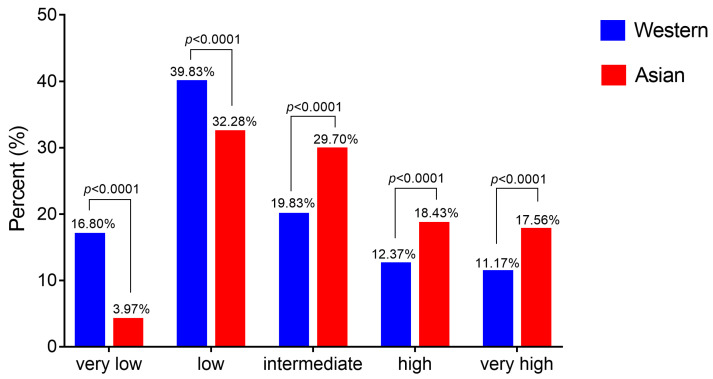
Distribution depending on IPSS-R in Western and Asian countries. The graph was drawn upon publications [26,29,31,36,39,41,42,43,44,45,46,47]. Raw data and statistical analysis are presented in Supplemental Table S4.

The International Prognostic Scoring System (IPSS), the WHO Prognostic Scoring System (WPSS), and the Revised-IPSS (IPSS-R) are the commonly used risk stratification systems for MDS patients. Among them, IPSS-R is well recommended for assessing prognosis because of its high evaluation effectiveness. It is based on five cytogenetic groups, in addition to the definitions of the depth of cytopenias and of bone marrow blast infiltration. For this reason, we only considered articles based on IPSS-R criteria [26,36,37,38,39]. Previous reports demonstrate that ethnic differences may exist in the prognosis of MDS patients in scoring category [28,40]. Here we collected and compared the prognosis scoring for MDS based on IPSS-R from different world areas [26,29,31,36,39,41,42,43,44,45,46,47] (Figure 3 and Figure 5, Table S4). Collectively, the sizes of Western and Asian cohorts are respectively 16,432 and 1458 patients (Table S4).

The IPSS-R classifies the risk among the following categories: very low, low, intermediate, high and very high risk. The IPSS-R is determined by combining the scores of 5 main features, including cytogenetics, bone marrow blasts, hemoglobin, platelets and absolute neutrophil count; among them, cytogenetics indicates the highest value. From the collected data (Figure 5), it appears that more patients are found in the low- and very low- risk categories in Europe and North America compared to Asian countries, and more patients are distributed in intermediate-, high- and very high- risk groups in Asia. 

Altogether, the data show that MDS is rare but at higher-risk in Asian population. We investigated the genetic factors to better understand these ethnic differences.

### 2.4. Genetic Characteristics Are Concordant with the Prognosis Difference Observed between Asian and Western MDS Patients

#### 2.4.1. Cytogenetics

**Figure 6:**
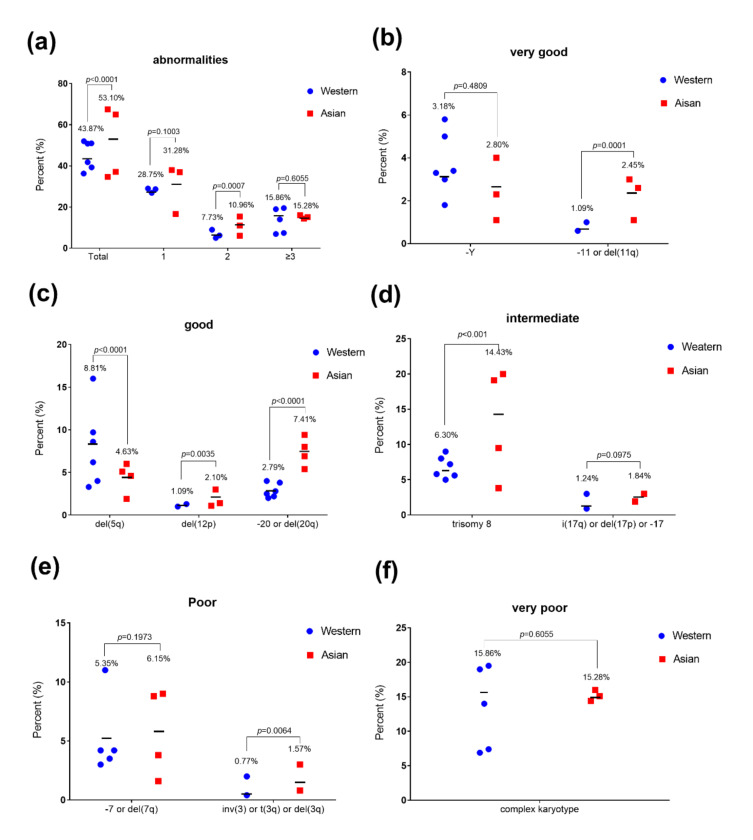
Karyotype distribution in Western and Asian MDS patients. (**a**) Percentage of total abnormalities, single abnormality, double abnormalities, 3 and more than 3 abnormalities. (**b**–**f**) Karyotype distribution in each cytogenetic risk group depending on IPSS-R. Because of the different sources of the data, we did not list the results completely consistent with IPSS-R, -11/del(11q) instead del(11q) in very good group, -20/del(20q) instead del(20q) in good group, i(17q)/del(17p)/-17 instead i(17q) in intermediate group, -7/del(7q) instead -7 in poor group and ≥3 abnormalities instead >3 abnormalities in very poor group. Figure 6 has been drawn based on the data and publications [8,26,29,31,32,48,49,50,51] described in Supplemental Tables S4 and S5.

Since the size of cohorts of recent MDS cytogenetic reports is small, we mainly concentrated our comparison on data collected a decade ago (Figure 3). Data are presented in Figure 6 and Tables S5 and S6 [8,26,29,31,32,48,49,50,51]. Differences in recurrent cytogenetic abnormalities between Western and Asian countries are consistently reported [8,21,28,29,31,48]. As a whole, the rate of detection of abnormal karyotypes is slightly higher in Asian vs. Western MDS populations (53.10% vs. 43.87%, *p* < 0.0001) (Figure 6a). Karyotypes with 2 abnormalities are again slightly more frequently reported in Asian compared to Western MDS population (10.96% vs. 7.73%, *p* = 0.0007). However, we do not observe significant differences in the rate of detection of karyotypes with 1 or more than 3 abnormalities in Asian compared to Western MDS populations (Figure 6a).

*Cytogenetics associated with very good prognosis:* -Y, -11 or del(11q). Chromosomal abnormalities -Y, -11 or del(11q) are associated with very good prognosis. There is no important difference in the rate of Y loss between Asian and Western populations (Figure 6b). On the contrary, the -11 or del(11q) abnormalities are significantly more observed in the Asian population compared to Western MDS patients (2.45%, vs. 1.09% *p* = 0.0001).

*Cytogenetics associated with good prognosis:* del(5q), del(12p), -20 or del(20q). Data show that the del(5q) chromosomal aberration is twice less frequently reported in the Asian MDS patients compared to the Western MDS patients (4.63% vs. 8.81%, *p* < 0.0001) (Figure 6c). In particular, MDS with isolated del(5q) is less frequent in Asian countries compared to Western countries (Tables S5 and S6). MDS with isolated del(5q) is considered to have a good prognosis, with a low probability of progression to secondary acute myeloid leukemia (AML) and longer life expectancy. The median survival of the Western patients with MDS with isolated del(5q) is 80 months, but decreases dramatically when additional karyotype abnormalities are present [32]. Unexpectedly, the Chinese median survival is 62 months in patients with isolated del(5q) and 78 months in patients with del(5q) plus one additional cytogenetic abnormality except monosomy 7 or del (7q) [52]. The rate of detection of del(12p) is slightly higher in Asian compared to Western MDS population (2.10% vs. 1.09%, *p* = 0.0035) (Figure 6c). Importantly, the del(20q) abnormality is more than twice frequently observed in the Asian patients (7.41% vs. 2.79%, *p* < 0.0001) (Figure 6c). MDS with isolated del(20q) show lower platelet counts, lower marrow blast counts, higher reticulocyte counts, and is associated with a favorable prognosis with a median survival of 54–71 months, and 12–15 months for del(20q) with additional abnormalities [32,53,54]. The AML progression rate is 14% for patients with isolated del(20q), 11% with one and 24% with several additional abnormalities in the Western MDS population [53]. However, in a Japanese study [55], the median survival of MDS patients with sole del(20q) is 80 months and 23 months when del(20q) is not the sole anomaly. Furthermore, del(20q) abnormality develops as a minor clone in the late stages of MDS indicating a clonal evolution toward leukemia, and poor prognosis. Accumulated genetic damage and genetic instability are thought to be the reasons [55].

*Cytogenetics associated with intermediate prognosis:* trisomy 8, i(17q), del(17p) or -17. The average rate of trisomy 8 in the Asian MDS population is 14.43%, whereas the average rate of trisomy 8 in the Western MDS population is down to 6.30%. This difference is significant (*p* < 0.001) but with a range of values in the Asian dataset ranging from 3.8% to 20% (Figure 6d). Trisomy 8 is observed in all the MDS subtypes, and isolated trisomy 8 is included in the IPSS-R intermediate cytogenetic risk group [41]. According to Western reports, the median survival of isolated +8 is 22–34.3 months, but increases to 40–44 months with 1 additional abnormality, and decreases to 23.8 months with 2 additional abnormalities, and to 5.8 months with three or more aberrations [32,56]. However, a Chinese report also indicated an association of isolated trisomy 8 with a much better prognosis, and a median survival of 44 months [29], which was higher than the Western results. A previous study disclosed that a high frequency of myeloproliferative features either at diagnosis or during evolution in MDS with isolated +8, respond poorly to hypomethylating agents (HMAs) [57]. Moreover, patients with isolated trisomy 8 present a high risk of progression to AML [26]. However immunosuppressive therapy response rate of MDS with +8 was significant [58]. The discrepancy in isolated trisomy 8 between Western and Asian countries should be verified in larger cohorts. Finally, no important differences is observed for the rate of i(17q), del(17p) or -17 between Western and Asian MDS populations (Figure 6d). They are rare and range from 0 to 3% in the different studies.

*Cytogenetics associated with poor prognosis:* -7, del(7q), inv(3), t(3q) or del(3q). We have also compared the distribution of -7 or del(7q) abnormalities, which correlate to a poor prognosis, and no significant differences were found between the Asian and the Western MDS populations (Figure 6e). Abnormalities on chromosome 3 including inv(3), t(3q) or del(3q) are also consistently reported in Western and Asian populations with a rate under 3%.

*Cytogenetics associated with very poor prognosis.* The distribution of the complex karyotype (>3 chromosomal abnormalities), which correlates to a very poor prognosis, does not show a significant difference between the Asian and the Western MDS populations (Figure 6a, enlarged in Figure 6f). The average percentage of detection of complex karyotypes is about 15% both in Asian and Western MDS populations. Additional publications showed that other abnormal karyotypes including +1/+1q, -1/del(1q), der(1;7), -9/del(9q) and del(16q) are significantly increased in Japanese compared to Caucasian populations [31], and a good prognostic impact is identified in +1/+1q, der(1;7),and del(9q) abnormalities [32,59]. The cytogenetic abnormalities play an important role on prognosis; the IPSS-R cytogenetic categories have discounted some rare karyotypes with independent prognostic values. Due to the profound cytogenetic heterogeneity of MDS, further investigation of the prognostic significances of many rare abnormalities is warranted.

Altogether, combining with cytogenetic data, we propose that the over-representation of MDS associated to del(5q) may account for the higher proportion of the low risk group in Western countries. Similarly, the higher proportion of trisomy 8 in Asian countries may explain the higher representation of patients in the intermediate risk category.

Considering age of onset, the median age of patients with trisomy 8 was younger than that of patients with del(5q) (43 vs. 59 years old). Patients with sole trisomy 8 were also younger than that with sole del(5q) (median age of 43 vs. 63 year-old) [29]. Therefore, the higher proportion of trisomy 8 observed in the Asian countries and the higher proportion of del(5q) observed in the Western countries are one of the plausible genetic factors explaining the discrepancy of almost two decades for the age of MDS onset in those countries.

#### 2.4.2. Molecular Genetics

**Figure 7:**
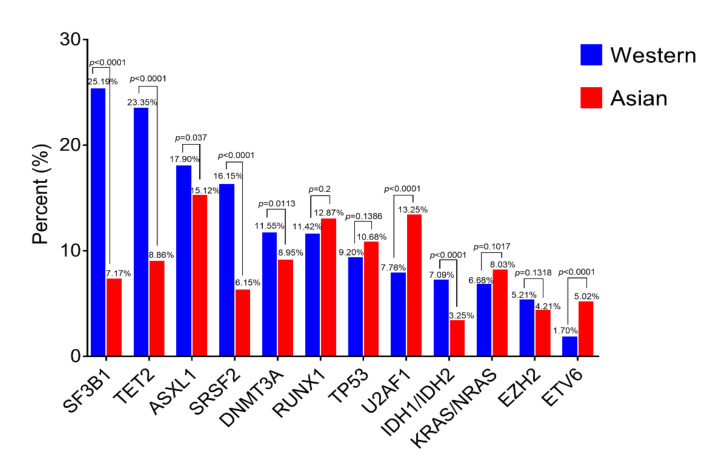
Gene mutation topographies in Western and Asian countries. Frequency of the most common gene mutations in Western and Asian countries. The data were analyzed upon 11 publications [30,62,63,67,68,69,70,71,72,73,74]. Raw data and statistical analysis are presented in Supplemental Table S7.

Gene mutations are thought to be acquired and positively selected to allow the expansion of the initiating clone to compromise normal hematopoiesis, and in due course give rise to MDS and subsequent transformation to AML [60]. Acquired mutations in genes involved in epigenetic regulation and chromatin remodeling (*TET2, DNMT3A, ASXL1, IDH1/2, EZH2*), pre-mRNA splicing (*SF3B1, SRSF2, U2AF1*), transcription (*TP53, RUNX1, ETV6*) and signaling transduction (*KRAS/NRAS*) are recurrently seen in most MDS [61,62,63,64], and some somatic mutations in certain genes can predict patient outcomes [65]. Different mutation topographies have been reported in hematological neoplasms between Asian and Western countries [66]; herein we analyzed the frequency of the most common 12 mutated genes in different countries [30,62,63,67,68,69,70,71,72,73,74] (Figure 3 and Figure 7, Table S7). Western and Asian mutations data include cohorts of respectively up to 2920 and 1790 patients (Table S7).

*Distribution of the gene mutations.* The gene mutation rate differs significantly among different studies, ranging from 51.5 to 89.5% in Western countries and from 55 to 91.4% in Asian countries. Genes with a mutation frequency greater than 10% are *SF3B1, TET2, ASXL1, SRSF2, DNMT3A* and *RUNX1* in Western countries, and *ASXL1, RUNX1, U2AF1* and *TP53* in Asia. The genes *IDH1/2, KRAS/NRAS, EZH2* and *ETV6* are mutated for less than 10% in MDS population, both in Asian and Western countries (Figure 7, Table S7).

In particular, *SF3B1*, *TET2, SRSF2* and *IDH1/2* are reported mutated three-time less in Asia compared to the Western MDS population (*SF3B1:* 7.17% vs. 25.19%; *SRSF2:* 6.15% vs. 16.15%; *TET2:* 8.86% vs. 23.35%; *IDH1/2*: 3.25% vs. 7.09%; *p* < 0.0001 for each gene). The *ASXL1* and *DNMT3A* genes are slightly less mutated in Asia compared to the Western MDS population (*ASXL1*: 15.12% vs. 17.90%, *p* < 0.05; *DNMT3A:* 8.95% vs. 11.55%, *p* < 0.05). On the contrary, *U2AF1* and *ETV6* are reported mutated twice as often among Asians (*U2AF1*: 13.25% vs. 7.76%, *p* < 0.0001; *ETV6:* 5.02% vs. 1.70%, *p* < 0.0001). Finally, the *RUNX1, TP53, KRAS/NRAS* and *EZH2* genes present equivalent mutation rates (*RUNX1:* ~12%; *TP53:* ~10%; *KRAS/NRAS:* ~7%; *EZH2:* ~5%, in Asian and Western populations respectively).

*Prognosis associated to gene mutation.* No systematic studies have been conducted to compare the prognosis of these mutations between the two parts of the world. *TET2* and *SF3B1* mutations are independent favorable prognostic factors [75,76,77]. On the contrary, *SRSF2* [78,79,80], *ASXL1* [81], *RUNX1* [82,83], *DNMT3A* [84,85], *U2AF1* [86], *TP53* [87,88,89,90], *EZH2* [91,92], *IDH1/2* [93,94,95,96], *KRAS/NRAS* [97,98] and *ETV6* [99] mutations are all associated with poorer clinical outcomes. 

Gene mutations are related to the therapeutic response. *TET2* and *DNMT3A* mutations predict a higher response rate to hypomethylating agents in MDS patients [100,101,102,103]. *RUNX1* and *TP53* mutations are associated with significantly lower responses to hypomethylating treatment, and *TP53* mutations show a high risk of relapse after allogeneic hematopoietic cell transplantation [81,87,88,89,90]. A Korean study described that *U2AF1* mutation does not affect the response rate to first-line decitabine treatment [104]. The presence of an *SF3B1* mutation adversely influences response to immunosuppressive therapy [105]. *TP53* mutations prove to have a negative impact on sensitivity to lenalidomide in MDS with isolated del(5q) [106].

However, new target therapies may improve the clinical prognosis of these gene mutations. For example, spliceosome modulators may be a therapeutic window of MDS with *SF3B1* mutations [107]. In this case, Western population may be more concerned by this therapy than Asian population because *SF3B1* is more frequently mutated in Western MDS patients rather that in Asia. Small molecule inhibitor APR-246 seems to improve the prognosis of *TP53* mutation MDS both as a single agent as well as in combination with AZA [6,108]. Ivosidenib and enasidenib may be an effective option for *IDH1* and *IDH2* mutations [5,109]. Less MDS patients are affected by *IDH1* and *IDH2* mutations in Asia than in Western countries. Pre-mRNA splicing modulators like sudemycins demonstrate a potential effect for treating hematological cancers harboring *U2AF1* mutations [110].

The relationships between gene mutations and gender, age and cytogenetics are also reported. An Asian study demonstrated that *SRSF2* mutation is closely associated with male sex and older age, and the prognostic impact of *SRSF2* mutation might be attributed to its close association with old age [80]. *TP53* mutations in MDS are strongly correlated with childhood and therapy-related MDS [87,111,112]. *U2AF1* mutation is more prevalent in younger MDS patients [86], and the *U2AF1* mutation is strongly associated with isolated trisomy 8 and del(20q) in Asian people, but not in Caucasian people, and is characterized by a younger age of MDS onset (median 39 year-old) [113]. 

MDS is typically present in older adults with the acquisition of age-related somatic mutations, whereas MDS present in children and younger adults is more frequently associated with germline genetic predisposition [114]. Considering the significant difference of onset age between Western and Asian populations, Yu et al. compared the difference of gene mutation between younger groups (16~59 years) and older groups (60~87 years) of MDS patients; they reported a trend towards higher incidence of gene mutations in the older group [115]. 

Altogether, cytogenetic aberrations appear to be more frequent in some ethnic groups, as well as some gene mutations. The reasons explaining those observations are not clear. The genetic differences observed between Western and Asian populations are relatively consistent with the distribution of MDS subtypes and the age of MDS onset. A higher frequency of *SF3B1* mutation is concordantly associated with a higher proportion of MDS with ring sideroblasts (MDS-RS) in Western countries. *TET2* is an age-associated mutation found in normal elderly individuals with acquired clonal hematopoiesis [116,117]. TET2 inactivation results in clonal hematopoiesis of indeterminate potential (CHIP) and favors transformation to myeloproliferative and myelodysplastic neoplasms (MPN, MDS) and AML [117]. The fact that MDS appears in older people in Western rather that in Asian population may explain that the rate of *TET2* mutation is higher in Western MDS population. Moreover, MDS, like other cancers, is considered to emerge after successive positive selections, where gene mutations and genetic alterations are central players [60]. We propose that specific ethnic variants or combination of variants will differently favor a positive selection of the initiating clone in a permissive microenvironment with specific sets of mutations.

Importantly, Asian MDS patients show more frequently high-risk cytogenetic aberrations. Therefore, we next question the survival of patients.

### 2.5. Survival Rates Are Equivalent for Asian and Western MDS Patients and Strictly Correlated to the Prognostic Groups

#### 2.5.1. Therapeutic Options

A variety of risk-adapted treatment strategies are adopted by clinicians due to the heterogeneity of MDS. The MDS International Working Group (IWG) puts forward the response criteria to treatment in 2000 and further revised it in 2006 to make the results of different clinical treatments comparable. Allogeneic hematopoietic stem cell transplantation (HSCT) is currently the only potentially curative option. Over the past two decades, azacitidine, lenalidomide, decitabine, deferasirox, epoetin alpha and luspatercept have gained market authorization from the US Food and Drug Administration (FDA) and the European Medicines Agency (EMA) for MDS. Among them, azacitidine, lenalidomide and decitabine are considered to modify the disease course. Epoetin alpha and luspatercept improve cytopenia. Deferasirox, a major oral iron chelator, reduces chronic iron overload in patients who are receiving long-term blood transfusions. The exact impact of transfusional systemic iron overload in MDS to AML transformation is still under question [118]. However, as far as we know, there is currently no systematic study comparing the deferasirox treatment response rates between Asian and Western countries. Treatment approach differences still exist in Western and Asian countries due to the discrepancy of MDS onset age, cytogenetics and prognostic groups.

The therapeutic response rate of lenalidomide is higher in patients with del(5q) (83%) compared to patients with normal karyotypes (57%) and other chromosomal abnormalities (12%). In addition, in intermediate-2 or high-risk patients with isolated del(5q), lenalidomide was proved to have a much better complete response rate than patients with one more additional cytogenetic abnormality [3]. Lenalidomide has been recommended by the National Comprehensive Cancer Network (NCCN) in the United States as the first-choice treatment for patients with del(5q) chromosomal abnormalities alone or with an additional cytogenetic abnormality, except those involving chromosome 7. Del(5q) is less frequently detected in the Asian population, and few studies have been conducted on lenalidomide treatment on MDS del(5q) in this population. Clinical trials implicating small size cohorts from China and Japan showed that the therapeutic response rate for 5q- syndrome is comparable to that in Europe and the USA [52,119]. However, the MDS patients with del(5q) in Asian countries are mostly accompanied by complex abnormalities [29].

Hypomethylating therapy using 5-azacitidine and 5-aza-2-deoxycytidine (decitabine) can be proposed to patients with MDS in higher-risk groups. Compared with supportive treatment groups, it can reduce the risk of patients progressing to AML and can improve survival. A Chinese report showed that azacitidine emerges as an effective and safe treatment strategy for Chinese patients with high-risk MDS, and therapeutic response rates are comparable to those from the Western patients in the phase 3 AZA-001 study [120]. A prospective study (DIVA) demonstrated that the effectiveness of hypomethylating agents in Korean patients is similar to those of the American multi-centre, phase II, clinical trial (ADOPT) [121]. Another study enrolling 135 Chinese cases with a median age of 54.1 years, all having *de novo* MDS and the majority of intermediate-1 or intermediate-2 IPSS, showed the complete response rate to be higher (66.7%) compared to that in the ADOPT study (52%) [122]. However, the complete response rate of a Japanese study was lower (32.4%) [123]. The difference in the baseline clinical characteristics of patients and number of treatment cycles may result in the disparity. An international, multi-centre, prospective clinical trial may help explain these differences.

As the only curative strategy, HSCT is recommended for younger MDS patients in higher risk groups and lower risk groups with poor prognosis genetic abnormalities. Despite its curative potential, the treatment-related mortality and the risk of relapse, age and comorbidities turn out to be a serious obstacle for elderly patients [124]. Albeit reduced-intensity conditioning transplantation offers overall and quality-adjusted survival benefit for patients with de novo MDS aged 60 to 70 years old in intermediate-2/high IPSS [125], post-HCT relapse still should be taken into account [126]. MDS patients being usually elderly with a median age of approximately 75 years at diagnosis in Western countries, compared to 60 years of age in Asian countries; transplant accessibility and the intensity of transplant conditioning are doomed to be different.

#### 2.5.2. Survival Rate

**Figure 8:**
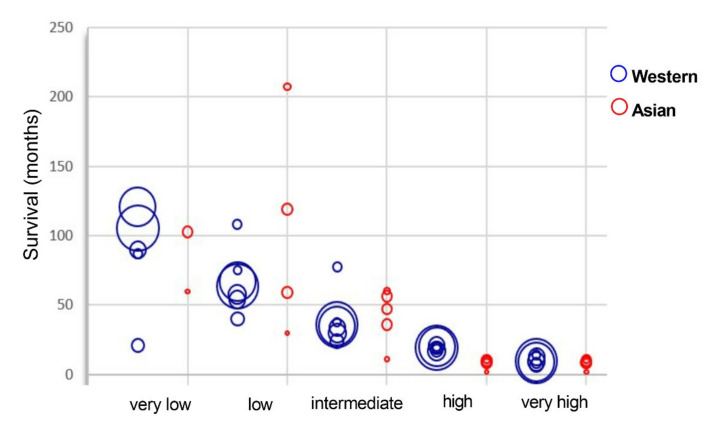
Survival depending on IPSS-R in Western and Asian countries. The size of the circles indicates the sample size of each study. The graph was drawn upon publications [26,29,31,36,40,42,43,44,45,46,47,48]. Raw data and statistical analyses are presented in Supplemental Table S8.

We collected and compared the survival data from different world areas [26,29,31,36,39,41,42,43,44,45,46,47] (Figure 3 and Figure 8, Table S8). 

The survival rate varies significantly among the studies, and we could not draw a clear difference between Asian and Western countries (Figure 8). However, a trend appears from regional reports. The prognostic discrimination of IPSS-R for survival is shown in most risk groups, but for the very low and low risk categories, some studies show an even longer survival for the low risk group than the very low risk group. Additional significant factors for predicting survival, including performance status, serum ferritin levels, lactate dehydrogenase levels, chronic comorbidity conditions, and mutations are thought to be the causes of this unexpected result [26,44,45,46]. Among them, gene mutations such as *TP53* are important factors explaining poor prognosis in patients at low risk category [26,127]. Furthermore, geriatric and male gender are also associated with reduced overall survival and more advanced leukemia [41,42,48,128].

Akira Matsuda et al. compared the overall survival (OS) of Japanese patients with refractory anemia in French-American-British (FAB) classification to that of German patients. Japanese patients show significantly more favorable prognosis for patients aged 60 years or younger. For patients older than 60 years, no favorable OS is shown [7]. In another study comparing clinical features of Japanese and Caucasian MDS patients, a striking difference is found in OS but not in time to AML transformation. The significantly improved survival in Japanese populations holds even after considering age, FAB and IPSS-R categories. The difference in OS mainly comes from lower-risk IPSS-R categories, including very low, low, and intermediate risk groups [31]. About AML transformation rate, the way of presenting this rate varies a lot from one study to another, so we could not get enough comparable data to perform a relevant comparison of the AML transformation rate between the Western and Asian populations. One study presenting the AML transformation rate between the Japanese and Caucasian, did not identify any significant difference [31].

## 3. Materials and Methods 

**Selection of studies.** We performed a literature search in PubMed and Google Scholar over the past two decades, with no restriction to language of publication. MESH terms and exclusion criteria are indicated in Figure 1 and Figure 3. For incidence and epidemiology data, we also considered the national cancer registries of reference with available published results. We finally considered 10 articles (Figure 1). For classification data, and due to successive modifications of diagnosis criteria [1], the studies based on World Health Organization (WHO) 2008 classification system were included, therefore restricted to 2008–2020. Additionally, we manually checked all the articles and excluded the studies based on FAB classification (Figure 3). For prognostic studies, we collected data under the Revised International Prognostic Scoring System (IPSS-R) so restricted to 2012–2020. In total, we retrieved 14 studies for the subtype distribution, 12 for the risk stratification, 9 for cytogenetics, and 11 for gene mutations (Figure 3). 

**Statistical analysis.***Incidence.* We calculated the weighted crude incidence for each study. Pooled incidence estimates were calculated with MetaXL version 5.3 software (Epigear International) using a quality-effects model by area. The quality scores of all the studies included into the analysis were more than 8 reflecting the very high quality of each study. Heterogeneity between studies was assessed with a X^2^ test (Cochran Q statistic) and quantified with the I^2^ statistic. Differences of incidence between the Western and Asian datasets were calculated using *t*-tests. *Classifications, cytogenetics, subtype distribution and prognosis*. We performed weighted analyses. The differences of count data between the Western and Asian datasets were calculated using contingency tables and X^2^ tests (GraphPad Prism version 7.0). A *p*-value less than 0.05 is considered statistically significant.

**Geographic criteria.** We refer to Asian population for China, Korea, and Japan. Western countries are more representative of Caucasian population. They consist of USA, Canada, Argentina (or America) and Netherland, Switzerland, France, Italy, Germany, Greece, Austria, Sweden, Poland (or Europe), and Australia because of the high representation of inhabitants from European origin in this country. 

## 4. Conclusions

MDS is a group of very heterogeneous syndromes. Here, we show that both differences and similarities exist in epidemiology, clinical features, genetics, and prognosis between Western and Asian countries (Graphical Abstract). In the era of precision medicine, those ethnic specificities should be taken into account for drug development. 

Compared to the Western MDS population, Asian MDS patients show a 2- to 4-fold lower incidence and up to 10 years of earlier onset age. Analyzing the causes of this early onset in Asia will be of great interest and may be beneficial to understand the pathogenesis of younger Western MDS patients. The subtypes MDS-RS, MDS-del(5q) and MDS-U are more represented in Western countries whereas MDS-SLD, MDS-MLD and MDS-EB are more represented in Asian countries. 

Genetic characteristics present subtle differences between Western and Asian MDS population. As for the cytogenetics abnormalities, the del(5q) is more frequently found in Western compared to Asian MDS population, whilst trisomy 8 and del(20q) are more frequently identified in Asian compared to the Western MDS population. The reasons of such cytogenetic differences remain unknown but this genetic specificity may contribute to explain the severity of MDS in Asian population. The rate of mutation is found half that of the Western for four genes, *TET2, SF3B1*, *SRSF2* and *IDH1/2* in the Asian MDS population, while, *U2AF1* and *ETV6* are more frequently reported in Asian MDS patients. Mutation rates are equally identified among the other major reported mutated genes of MDS, *ASXL1, RUNX1, DNMT3A, TP53* (with a frequency of more than or close to 10% each), and in *EZH2* and *KRAS/NRAS* (with a mutation frequency of about 5%).

Meanwhile, very low- and low-risk groups of IPSS-R are reported in Western MDS population whereas the intermediate, high and very high-risk groups are more represented in Asian MDS population. The above differences lead to disparities in therapeutic effects of immunomodulation, immunosuppression, hypomethylating reagents, as well as accessibility and conditioning of hematopoietic stem cell transplantation. Finally, we did not show substantial differences for the survival rate within each prognostic group for MDS patients between Western and Asian countries.

Altogether, systematic research to analyze and compare the molecular, genetic, and environmental differences among different regions of the world may open a window for a better understanding of MDS pathogenesis. This may lead to the design of personalized tailored treatments adapted to the diversity of the world populations.

## Data Availability

Publicly available datasets were analyzed in this study. This data can be found in each publication that we have referenced.

## Supplementary Materials

The following are available online at https://www.mdpi.com/2072-6694/13/3/481/s1, Table S1. Studies comparing the epidemiological difference between Western an Asian MDS patients. Table S2. Studies reporting the percentage of MDS subtypes according to WHO 2008 and ICD-O classification. Table S3. Raw data and statistical analysis of MDS subtypes according to different areas in the world. Table S4. Raw data and statistical analysis of MDS IPSS-R distribution according to different areas in the world. Table S5. Studies reporting cytogenetic characteristics in MDS. Table S6. Raw data and statistical analysis of recurrent cytogenetic abnormalities in MDS according to different areas in the world. The tables are separately presented according to the cytogenetic score. Table S7. Raw data and statistical analysis of recurrently mutated genes in MDS according to different areas in the world. Table S8. Raw data of MDS median overall survival in month and distribution among each IPSS-R group.
