# Supplementary material for: Asian Population Is More Prone to Develop High-Risk Myelodysplastic Syndrome, Concordantly with Their Propensity to Exhibit High-Risk Cytogenetic Aberrations
Source: Cancers (Basel). 2021 Jan 27;13(3):481. doi: 10.3390/cancers13030481 (PMC7865620; doi:10.3390/cancers13030481)
Supplement: Supplementary file 1 [file cancers-13-00481-s001.pdf]

Table S1. Studies comparing the epidemiological difference between Western and Asian MDS patients.

	Age-adjusted incidence (/10^5)												
	Total	Gender		age						Median age	Resource	Reference	Year
		Male	Female	0-40y	40-49y	50-59y	60-69y	70-79y	>80y				
USA	3.27	4.43	2.53	0.14	0.62	1.95	7.14	20.05	35.49	-	NAACCR&SEER	Rollison DE 2008	2001-2003
	7	7.7	6.2		0.9(<50y)	2.2	15	40.8	82.9	-	GHC	Roos AJD 2010	2005-2006
	4.9	6.7	3.8	0.2	0.8	2.5	9.9	30.3	58	-	SEER	SEER 2011	2007-2011
	4.5	6.2	3.3	0.1	0.7	2.1	8.2	26.9	55.4	-	SEER	SEER 2016	2012-2016
Netherlands	2.26	2.97	1.54		0.25(<50y)-	1.79	5.86	16.48	24.95	74	NCR	Dinmohamed AG 2014	2001-2005
	2.76	3.66	1.86		0.26(<50y)-	2.01	7.19	20.47	32.13	75	NCR		2006-2010
Switzerland	2.45	3.34	1.82			0.77(<65y)	10.33(65-74y)	25.56(75-84y)	27.34(>84y)	76	CCRs	Bonadies N 2017	2001-2007
	2.53	3.55	1.8			0.68(<65y)	11.34(65-74y)	26.45(75-84y)	33.53(>84y)	77	NICER		2008-2012
France	male	3.4		-	0.3(<50y)	1.3	5.8	24.4	74.2	78	FRANCIM	Réseau FRANCIM 2019	2018
	female		1.6	-	0.4(<50y)	1.1	2.9	11.2	30.2	80			
Japan		1.61	0.82				-			76	MCIJ	Chihara D 2014	2008
China	1.51	1.48	1.54	0.34(18-34y)		1.09(35-59y)		3.97(>60y)		62	Shanghai	Wang W 2012	2004-2007
Korea	1.13	male		0.13(0-14y)	0.35(15-34y)	0.81(35-49y)	2.44(50-64y)	11.88(65-79y)	19.97(>80y)	-	KCCR	Park E-H 2015	2012
		female		0.11(0-14y)	0.28(15-34y)	0.67(35-49y)	1.31(50-64y)	4.81(65-79y)	6.24(>80y)				

Abbreviations: NAACCR, the North American Association of Central Cancer Registries; SEER, the Surveillance, Epidemiology, and End Results; GHC, Group Health Cooperative; NCR, Netherlands Cancer Registry; CCRs, Swiss Cantonal Cancer Registries; NICER, The National Institute for Epidemiology and Cancer Registration; MCIJ, Monitoring of Cancer Incidence in Japan project; KCCR, The Korea Central Cancer Registry.

Table S2. Studies reporting the percentage of MDS subtypes according to WHO 2008 and ICD-O classification.

		ICD-O-3	USA Rollison DE 2008	USA SEER 2016	Switzerland- Bonadies N 2017	Nether-lands Dinmo-hamed AG 2014	Poland Madry K 2015	Sweden Berggren 2018	Australia McQuilten ZK 2014	Korea Park EH 2015	China Wang W 2012	China Wang H 2010	China Zhang TT 2016	China Qu SQ 2012	China Yao CY 2016	Japan Miyazaki 2018
MDS-SLD	RA	9980	14.4	5.3	3.4	8.5	19.7		12		12.3	2.3		11		
	RN	9991	-	-	0	-	-	9	-	20.9	-	-	9.1	0.4	20.3	16.8
	RT	9992	-	-	0.1	-	-		-		-	-		0.6		
MDS-RS		9982	10.0	5.8	3.8	11.8	6.7	11	6		4.7	1.1	5.1	4.0	5.4	4.0
MDS-EB-1		9983	13.0	16.3	17.5	20.2	13.9	17	11.5	43.8	15.3	9.7	19.8	20	21.1	10.6
MDS-EB-2							19.6	18				14.5	21.8	17	23.1	21.2
MDS-MLD		9985	2.4	7.9	10.6	14.6	30.4	30	15	34.2	57.6	69.6	29.5	43	29.3	41.2
MDS-5q		9986	1.7	3.7	2.4	2.3	4.6	4	1	1.1	3.4	0.5	0.9	1.0	-	1.3
t-MDS		9987	2.5	-	1.6	-	3.8	14	2	-	-	-	-	-	-	-
MDS-U		9989	56.1	61	60.7	42.6	5.1	11	52	-	6.8	2.3	13.8	3	0.8	4.9
Number of total case			24798	28138	1031	2981	863	1329	4254	7327	236	435	550	532	369	226
resource			NAACCR &SEER	SEER	NICER	NCR	Polish	Swedish MDS-register	VCR &VAED	KCCR	Shanghai	shanghai	Soochow	CAMS &PUMC	Taiwan	IWG-PM
time			2001-2003	2012-2016	2008-2012	2006-2010	2008-2009	2009-2013	2003-2010	2001-2012	2004-2007	2003-2007	2001-2013	1990-2010	1990-2010	1964-2010

Table S3. Raw data and statistical analysis of MDS subtypes according to different areas in the world. The data have been extracted from the publications described in supplemental Table S2.

	MDS-SLD		MDS-RS		MDS-EB		MDS-MLD		MDS-5q-		MDS-U	
	Western	Asia	Western	Asia	Western	Asia	Western	Asia	Western	Asia	Western	Asia
number of cases	6152	1797	4962	94	9836	4011	4662	3537	1691	104	35374	132
total cohort size	63394	9675	63394	9675	63394	9675	63394	9675	63394	9306	63394	2348
weighted percentage (%)	9.70%	18.57%	7.83%	0.97%	15.52%	41.46%	7.35%	36.56%	2.67%	1.12%	55.80%	5.62%
p-value (X^2 test)	<0.0001		<0.0001		<0.0001		<0.0001		<0.0001		<0.0001	

Table S4. Raw data and statistical analysis of MDS IPSS-R distribution according to different areas in the world.

	Western			Asia			
IPSS-R	number of cases	total cohort size	weighted percentage (%)	number of cases	total cohort size	weighted percentage (%)	*p* value (X^2 test)
very low	2760	16432	16.80	58	1458	3.97	<0.0001
low	6544	16432	39.83	471	1458	32.28	<0.0001
intermediate	3258	16432	19.83	560	1884	29.70	<0.0001
high	2033	16432	12.37	347	1884	18.43	<0.0001
very high	1835	16432	11.17	331	1884	17.56	<0.0001

Table S5. Studies reporting cytogenetic characteristics in MDS.

		Austria & Germany<br>Hasse D 2007	Germany & Austria & USA<br>Schanz J 2011	Sweden<br>Berggren DM 2018	Greece<br>Avgerinou C 2013	Argentina<br>Belli CB 2011	Caucasian<br>Miyazaki Y 2018	Japan<br>Miyazaki Y 2018	China<br>Qu SQ 2012	China<br>Li L 2019	China<br>Chen B 2005
karyotype	normal	988(48%)	1156(49.2%)	483(49%)	294(60.7%)	245(58.2%)	3660(62.7%)	196(65.3)	186(35%)	114(32.5%)	231（62.9%）
	abnormal	1084(52%)	1195(50.8%)	512(51%)	190(39.3%)	176(41.8%)	2178(36.3%)	104(34.7%)	346(65%)	237(67.5%)	136(37.1%)
Number of abnormal karyotypes	1	605(29%)	737(31.3%)	331(33%)	130(26.9%)	121(28.7%)	-	-	200(38%)	130(37%)	61(16.6%)
	2	180(9%)			24(5.0%)	26(6.2%)			61(11%)	54(15.4%)	22(6.0%)
	3	299(14%)	458(19.5%)	36(4%)	36(7.4%)	29(6.9%)			85(16%)	53(15.1%)	53(14.4%)
	>3			145(15%)							
Frequent chromosomal abnormalities	-Y	54(3%)	42(1.8%)	53(5%)	28(5.8%)	14(3.3%)	164(3.4%)	3(1.1%)	21(4%)	8(2.3%)	-
	-11/del(11q)	22(1%)	-	4(0)	-	-	60(0.6%)	3(1.1%)	16(3%)	9(2.6%)	-
	del(5q)	324(16%)	146(6.2%)	42(4%)	16(3.3%)	41(9.7%)	415(8.6%)	5(1.9%)	30(6%)	18(5.1%)	17(4.6%)
	-5	65(3%)	-	0	-	-	0	0	21(4%)		
	del(12p)	22(1%)	-	3(0)	-	-	61(1.3%)	3(1.1%)	16(3%)	5(1.4%)	-
	-20/del(20q)	76(4%)	52(2.2%)	21(2%)	12(2.5%)	16(3.8%)	135(2.8%)	18(6.9%)	41(8%)	33(9.4%)	20(5.4%)
	+8	173(8%)	132(5.6%)	45(5%)	35(7.2%)	38(9.0%)	280(5.8%)	10(3.8%)	106(20%)	67(19.1%)	35(9.5%)
	+19	0	-	2(0)	-	-	23(0.5%)	0	0	0	-
	i(17q)/del(17p)/-17	54(3%)	-	2(0)	-	-	42(0.9%)	5(1.9%)	16(3%)	0	-
	-7/del(7q)	227(11%)	99(4.2%)	26(3%)	17(3.5%)	-	206(4.2%)	10(3.8%)	46(9%)	31(8.8%)	6(1.6%)
	Inv(3)/t(3q)/del(3q)	43(2%)	-	1(0)	-	-	17(0.4%)	2(0.8%)	16(3%)	0	-
Total case		2072	2351	995	484	421	4844	261	532	351	367
Year		1964-2004	1972-2010	2009-2013	1990-2009	1982-2010	1964-2010	1964-2010	1990-2010	1990-2008	1990-2003

Table S6. Raw data and statistical analysis of recurrent cytogenetic abnormalities in MDS according to different areas in the world. The data have been extracted from the publications described in supplemental Table 4. The tables are separately presented according to the cytogenetic score.

Very good cytogenetic score

	monosomy Y		monosomy 11/del(11q)	
	Western	Asia	Western	Asia
number of cases	355	32	86	28
total cohort size	11167	1144	7911	1144
weighted average (%)	3.18%	2.80%	1.09%	2.45%
p-value (X^2 test)	0.4809		0.0001	

	total abnormalities		1 abnormality		2 abnormalities		3 and more abnormalities	
	Western	Asia	Western	Asia	Western	Asia	Western	Asia
number of cases	5335	823	856	391	230	137	1003	191
total cohort size	12161	1550	2977	1250	2977	1250	6323	1250
weighted average (%)	43.87%	53.10%	28.75%	31.28%	7.73%	10.96%	15.86%	15.28%
p-value (X^2 test)	<0.0001		0.1003		0.0007		0.6055	

Good cytogenetic score

	del(5q)		del(12p)		monosomy 20/del(20q)	
	Western	Asia	Western	Asia	Western	Asia
number of cases	984	70	86	24	312	112
total cohort size	11167	1511	7911	1144	11167	1511
weighted average (%)	8.81%	4.63%	1.09%	2.10%	2.79%	7.41%
p-value (X^2 test)	<0.0001		0.0035		<0.0001	

Intermediate cytogenetic score

	trisomy 8		i(17q)/del(17p)/monosomy 17	
	Western	Asia	Western	Asia
number of cases	703	218	98	21
total cohort size	11167	1511	7911	1144
weighted average (%)	6.30%	14.43%	1.24%	1.84%
p-value (X^2 test)	<0.0001		0.0975	

Poor cytogenetic score

	monosomy 7/del(7q)		inv(3)/t(3q)/del(3q)	
	Western	Asia	Western	Asia
number of cases	575	93	61	18
total cohort size	10746	1511	7911	1144
weighted average (%)	5.35%	6.15%	0.77%	1.57%
p-value (X^2 test)	0.1973		0.0064	

Very poor cytogenetic score

	complex karyotype	
	Western	Asia
number of cases	1003	191
total cohort size	6323	1250
weighted average (%)	15.86%	15.28%
p-value (X^2 test)	0.6055	

Table S7. Raw data and statistical analysis of recurrently mutated genes in MDS according to different areas in the world. The data were analysed upon 11 publications (C.-Y. Yao, 2016; E. Papaemmanuil, 2013; T. Haferlach, 2014; M. J. Walter, 2013; R. Bejar, 2011; G. Montalban-Bravo, 2018; Y. Yu, 2020; Y. Xu, 2017; A. Tefferi, 2017; M. G. Della Porta, 2016; T. Yoshizato, 2017).

	**Western**			**Asia**			
gene	Number of cases	total cohort size	Weighted percentage (%)	Number of cases	total cohort size	Weighted percentage (%)	*p* value (X^2 test)
SF3B1	596	2367	25.19	128	1790	7.17	<0.0001
TET2	682	2920	23.35	159	1790	8.86	<0.0001
ASXL1	523	2920	17.90	271	1790	15.12	0.037
SRSF2	365	2262	16.15	110	1790	6.15	<0.0001
DNMT3A	324	2806	11.55	160	1790	8.95	0.0113
RUNX1	334	2920	11.42	230	1790	12.87	0.2
TP53	269	2920	9.20	191	1790	10.68	0.1386
U2AF1	184	2367	7.76	237	1790	13.25	<0.0001
IDH1/IDH2	207	2920	7.09	58	1790	3.25	<0.0001
KRAS/NRAS	195	2920	6.68	144	1790	8.03	0.1017
EZH2	152	2920	5.21	75	1790	4.21	0.1318
ETV6	37	2197	1.70	71	1421	5.02	<0.0001

Table S8. Raw data of MDS median overall survival in month and distribution among each IPSS-R group.

	USA A Mishra 2013	USA N Gangat 2013	Europe MG Della Porta 2015	Europe J Kaivers 2018	Sweden D Moreno Berggren 2018	Italy MT Voso 2013	Europe&America PL Greenberg 2012	China SQ Qu 2012	China MY Du 2020	China-Taiwan YT Yang 2014	China-Taiwan HA Hou 2018	Japan Y Miyazaki 2018
very low	90(14%)	21(5%)	121(18%)	87.1(9.9%)	NR(6%)	NR(38%)	105.6(19%)	NR(2%)	60(3.6%)	102.8(3.1%)	83.6(27.7%)	NR(10%)
low	54(32%)	40(63%)	67(37%)	108.3(39.6%)	57.7(56%)	75.1(33%)	63.6(38%)	59(43%)	30(29.1%)	118.9(22.9%)		207.6(31.7%)
intermediate	34(22%)	24(18%)	35(21%)	77.4(16.7%)	29.8(15%)	37.7(18%)	36(20%)	36(36%)	11(30.1%)	56.1(25.8%)	47.2(25.4%)	60(32%)
high	21(18%)	18(4%)	20(13%)	20.3(12.8%)	17(8%)	18.4(7%)	19.2(13%)	15(7%)	5(23.6%)	19.1(25.8%)	17.7(24.9%)	25.2(13.3%)
very high	13(14%)	6.5(10%)	9(11%)	8.2(20.9%)	9.3(15%)	14(4%)	9.6(10%)	10(12%)	2(12.7%)	8.4(22.5%)	7.8(22.1%)	10.8(13%)
case number	1158	783	5326	444	1329	380	7012	532	110	555	426	261

Abbreviations：NR, not reached.
